# A One-Health Perspective of Antimicrobial Resistance (AMR): Human, Animals and Environmental Health

**DOI:** 10.3390/life15101598

**Published:** 2025-10-13

**Authors:** Hanan Al-Khalaifah, Muhammad H. Rahman, Tahani Al-Surrayai, Ahmad Al-Dhumair, Mohammad Al-Hasan

**Affiliations:** Environment and Life Sciences Research Center, Kuwait Institute for Scientific Research, P.O. Box 24885, Kuwait City 13109, Kuwaitmhasan@kisr.edu.kw (M.A.-H.)

**Keywords:** AMR hotspots, environmental contamination, infection control, resistant bacteria, public health

## Abstract

Antibiotics are essential for treating bacterial and fungal infections in plants, animals, and humans. Their widespread use in agriculture and the food industry has significantly enhanced animal health and productivity. However, extensive and often inappropriate antibiotic use has driven the emergence and spread of antimicrobial resistance (AMR), a global health crisis marked by the reduced efficacy of antimicrobial treatments. Recognized by the World Health Organization (WHO) as one of the top ten global public health threats, AMR arises when certain bacteria harbor antimicrobial resistance genes (ARGs) that confer resistance that can be horizontally transferred to other bacteria, accelerating resistance spread in the environment. AMR poses a significant global health challenge, affecting humans, animals, and the environment alike. A One-Health perspective highlights the interconnected nature of these domains, emphasizing that resistant microorganisms spread across healthcare, agriculture, and the environment. Recent scientific advances such as metagenomic sequencing for resistance surveillance, innovative wastewater treatment technologies (e.g., ozonation, UV, membrane filtration), and the development of vaccines and probiotics as alternatives to antibiotics in livestock are helping to mitigate resistance. At the policy level, global initiatives including the WHO Global Action Plan on AMR, coordinated efforts by (Food and Agriculture Organization) FAO and World Organisation for Animal Health (WOAH), and recommendations from the O’Neill Report underscore the urgent need for international collaboration and sustainable interventions. By integrating these scientific and policy responses within the One-Health framework, stakeholders can improve antibiotic stewardship, reduce environmental contamination, and safeguard effective treatments for the future.

## 1. Introduction

AMR has emerged as one of the most critical global health threats, undermining the treatment of infectious diseases across humans, animals, and the environment. In 2019, AMR was linked to approximately 5 million deaths, with 1.27 million of these deaths directly attributable to drug-resistant bacterial infections. These deaths were caused by infections where AMR led to treatment failure, including bloodstream infections, pneumonia, tuberculosis, and intra-abdominal infections [1,2]. These deaths disproportionately affect low- and middle-income countries (LMICs), where inadequate surveillance, limited access to new antimicrobials, and insufficient healthcare infrastructure exacerbate the problem. Resistance to commonly used antibiotics, such as fluoroquinolones and beta-lactams, accounted for more than 70% of these deaths, underscoring the urgent need for coordinated action to combat AMR [2]. If current trends continue, AMR could lead to up to 10 million deaths annually by 2050, with profound consequences for public health and the global economy [3].

LMICs bear the greatest burden of AMR, with the highest mortality rates and economic consequences. Inadequate healthcare infrastructure, weak surveillance systems, and limited access to newer antimicrobials exacerbate the problem. Patients in LMICs often face delays in diagnosis and treatment, leading to prolonged infections and increased transmission of resistant pathogens. The widespread availability of antibiotics without prescription, coupled with poor infection prevention and control in healthcare facilities, accelerates resistance emergence.

Agriculture and aquaculture in LMICs also contribute significantly to AMR due to the unregulated use of antimicrobials for prophylaxis and growth promotion. Moreover, untreated pharmaceutical and municipal wastewater frequently contaminates rivers and soils, creating “resistance hotspots”. These conditions not only amplify the local spread of resistant organisms but also facilitate their global dissemination through trade, migration, and environmental flows.

AMR is driven by the overuse and misuse of antibiotics in human healthcare, animal farming, and agriculture. In healthcare settings, inappropriate prescribing, poor infection control practices, and the lack of effective diagnostics have led to the rapid rise of multidrug-resistant organisms, including methicillin-resistant * Staphylococcus aureus* (MRSA), carbapenem-resistant *Enterobacteriaceae* (CRE), and multidrug-resistant *Klebsiella pneumoniae* [2,4]. In animal husbandry, antibiotics are often used for growth promotion and disease prevention, creating reservoirs of resistant bacteria that can spread through direct contact or the food chain [5]. Similarly, agricultural practices, such as the use of antimicrobials in crops and fertilizers, contribute to the enrichment of resistance genes in the environment, contaminating soils and water [2].

**Figure 1:**
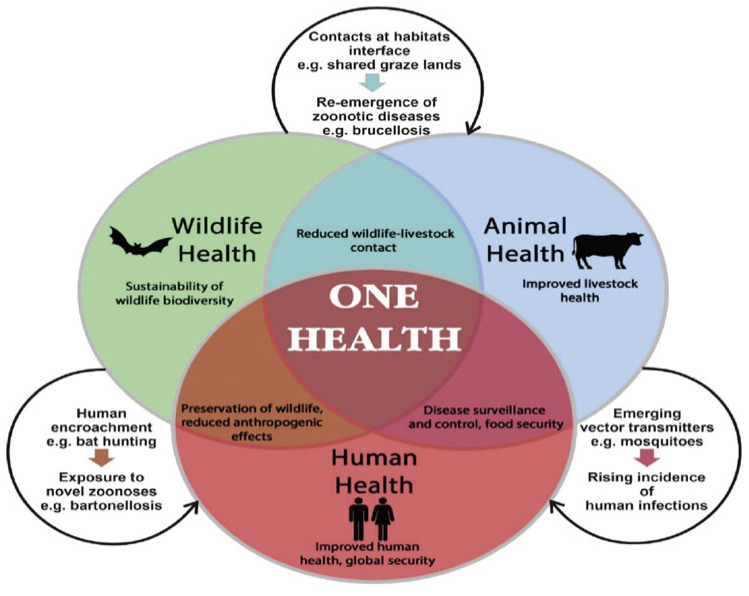
Schematic representation of the One Health concept [11].

Environmental contamination is also a key driver of AMR. Wastewater from hospitals, aquaculture systems, and agricultural runoff carries antibiotic residues and resistant bacteria into rivers, lakes, and marine sediments, promoting the spread of resistance genes and facilitating horizontal gene transfer (HGT) [6,7]. These environmental reservoirs create pathways for resistant pathogens to circulate back into humans and animals, highlighting the interconnected nature of AMR across human, animal, and environmental health (Figure 1). The One Health approach integrating human, animal, and environmental health has been advocated by the WHO and other global health bodies as a unified strategy to address this complex challenge.

Despite its importance, the environmental aspects of AMR, such as the role of wastewater and marine sediments, have received significant attention, while the contributions of human and animal health to AMR development need further emphasis. In human healthcare, resistance to critical antibiotics, such as those used for treating *Escherichia coli* infections, pneumonia, and tuberculosis, continues to increase. Similarly, the overuse of antibiotics in animal health, especially in poultry and livestock, has been shown to contribute to the spread of resistance. For instance, resistance genes shared between animals, humans, and the environment, such as macA-macB and tetA-tetR), have been identified in both animal feces and human pathogens [8].

AMR is a growing concern in both dairy and poultry farming, where antibiotic use is prevalent. Studies have shown that both raw and pasteurized milk can harbor resistance genes, which pose a significant public health risk, particularly when transmitted through consumption of contaminated milk or meat [9,10]. Similarly, in poultry farming, antibiotics like β-lactams and tetracycline are used extensively, leading to alterations in the gut microbiota and the development of resistance in both animals and humans. In these sectors, better stewardship and surveillance practices are urgently needed to reduce the spread of AMR.

While significant progress has been made in understanding the global burden of AMR, gaps remain in our knowledge regarding the relative contributions of human, animal, and environmental sectors to resistance development. Current research has largely focused on individual sectors, often neglecting the interconnected pathways through which AMR spreads across these domains. This review aims to fill this gap by synthesizing existing knowledge across all three sectors, namely human, animal, and environmental health, under the One Health framework. By addressing the integrated nature of AMR, this review will highlight key reservoirs, transmission pathways, and strategies for coordinated interventions. Specifically, it will emphasize the importance of surveillance, stewardship, and the need for global cooperation to mitigate the spread of AMR across sectors.

In particular, this review will focus on identifying knowledge gaps in the understanding of environmental contributions to AMR, including the role of wastewater and marine contamination, while also considering human and animal health aspects. By providing a more balanced perspective, this review will offer insights into the complexity of AMR transmission and the need for comprehensive, sector-wide solutions.

**Figure 2:**
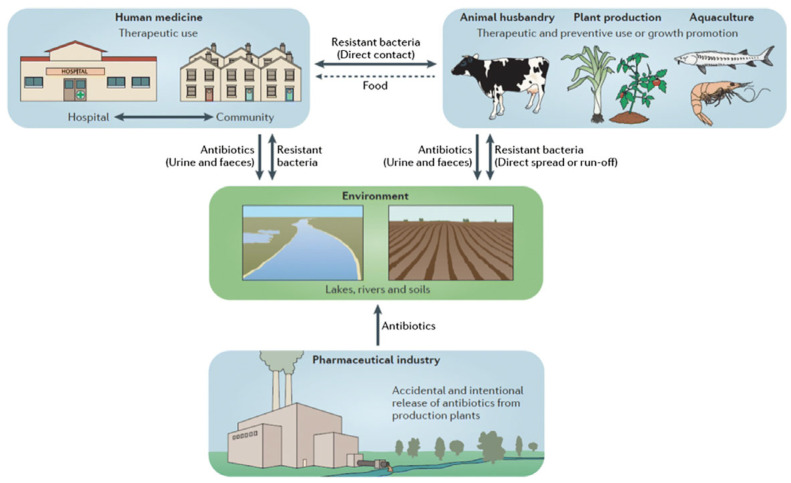
The Ecology of Resistance: A One-Health Perspective on the Spread of AMR [12].

As shown in Figure 2, resistance drivers are not sector specific; wastewater effluents, agricultural runoff, and foodborne transmission all create interconnected loops that facilitate ARGS persistence and HGT.

## 2. Methodology

This review provides a narrative synthesis of published studies on AMR from a One Health perspective, covering human, animal, and environmental health. Literature was identified through PubMed, Scopus, Web of Science, and Google Scholar, along with key reports from WHO, FAO, WOAH, and the O’Neill Report. We focused on English-language studies published between 2000 and 2025 that examined AMR drivers, pathways, surveillance, or interventions, with particular attention to the challenges faced in LMICs. Both peer-reviewed research articles and selected gray literature (e.g., WHO technical reports, CDC guidelines, and national policy frameworks) were included to capture surveillance and policy perspectives often underrepresented in academic literature.

### 2.1. AMR in Environmental Health: A One-Health Perspective

AMR is increasingly recognized as a significant environmental challenge, extending well beyond its traditional association with human and veterinary medicine. The natural environment functions as a critical reservoir, transmission route, and facilitator for the emergence, evolution, and dissemination of antimicrobial-resistant bacteria (ARB) and ARGs. In alignment with the One-Health approach which emphasizes the interconnectedness of human, animal, and environmental health, the role of the environment is now acknowledged as central to the dynamics of AMR [13].

The One-Health model draws attention to environmental transmission routes and ecological selection pressures that perpetuate resistance outside of host organisms. The environment enables HGT between microbial communities, fosters resistance persistence, and facilitates inter-compartmental transport of AMR through air, water, soil, and biotic vectors.

An environmental analysis of sewage, seawater, sediment, and aerosol samples documented widespread ARGs including those for extended-spectrum beta-lactamase (ESBL), carbapenemases, and colistin resistance. The cross-domain analysis also identified the overlapping of ARGs. Regarding the One-Health approach, none of the studies used this approach to interlink these sectors [14].

### 2.2. Climate Change as an Amplifier of AMR Within the One Health Framework

Climate change accelerates AMR by creating favorable conditions for the spread and persistence of resistant organisms and pathogens, through increased temperatures, altered rainfall, and extreme weather events. This is amplified by disruptions to water and sanitation, increased disease outbreaks, and higher demand for antibiotics, particularly in vulnerable LMICs. While the relationship is not fully causal, evidence shows increased temperatures directly impact bacterial growth and HGT, contributing to resistance development [15]. Conversely Flooding, cyclones, and dust storms spread antibiotic-resistant pathogens and antimicrobial residues from untreated sewage and animal waste into water bodies and the atmosphere, creating risks for human, animal, and environmental health. Integrating climate change into AMR policy is vital for anticipating new resistance patterns and improving resilience within the One Health framework [16].

### 2.3. Major Environmental Sources and Pathways of AMR

The environment serves as a critical reservoir and transmission pathway for AMR, connecting human and animal health through water, soil, air, and food systems. Instead of isolated sources, AMR drivers can be broadly categorized into industrial and agricultural sources, municipal and wastewater sources, and natural ecosystems and wildlife.

#### 2.3.1. AMR in Pharmaceutical Industry Effluents

The pharmaceutical industry plays a crucial, yet often overlooked, role in the global AMR crisis. Within the One-Health framework, which emphasizes the interconnection between human, animal, and environmental health, industrial discharges from antibiotic manufacturing facilities represent a major environmental source and amplifier of ARB and ARGs. These effluents are particularly significant because they introduce exceptionally high concentrations of active pharmaceutical ingredients (APIs) directly into the environment, fostering intense selective pressure that favors the emergence and proliferation of resistant microbial populations.

Contaminated rivers and irrigation waters downstream from pharmaceutical zones have been found to harbor ARGs associated with resistance to β-lactams, macrolides, tetracycline, and fluoroquinolones, among others [17]. These genes persist in aquatic sediments, are taken up by environmental microbes, and can re-enter the human food chain via crops irrigated with contaminated water or fish raised in polluted ponds.

In many antibiotic-producing regions, especially in LMICs, pharmaceutical manufacturing effluents are often discharged untreated or partially treated into rivers, lakes, and soil systems. For example, studies conducted near industrial hubs such as Patancheru, India, revealed extremely high levels of ciprofloxacin (up to 31 mg/L) in treatment plant effluent, a concentration hundreds of times greater than levels needed to inhibit susceptible bacteria. These “resistance hotspots” facilitate the selection and enrichment of multidrug-resistant organisms, even in native aquatic or soil microbial communities.

In response to such environmental contamination, regulatory actions have emerged. India implemented the Environment (Protection) Amendment Rules in 2019, setting permissible antibiotic residue limits in effluents discharged by pharmaceutical manufacturing units [18]. This policy represents one of the first national efforts globally to regulate antibiotic pollution from industrial sources. Additionally, the Strategic Approach to International Chemicals Management (SAICM) provides a voluntary global framework to promote safer chemical management, including pharmaceuticals, encouraging nations to integrate chemical pollution controls into sustainable development goals [19].

From a One-Health perspective, the pharmaceutical industry’s role in environmental AMR highlights the interconnectedness of resistance development across human, animal, and environmental sectors. Resistant bacteria and ARGs from industrial waste can enter human populations through drinking water, recreational contact, or food; affect animals via contaminated feed or water; and persist in environmental reservoirs that facilitate ARG spread.

Addressing AMR through the One-Health lens necessitates the inclusion of industrial pollution control as a core component of national and international AMR action plans, WHO Global Action Plan, UK’s 2024–2029 plan, and Kuwait’s 2022 plan recognize the environmental dimension of AMR, explicit, enforceable regulations on industrial discharge are needed to strengthen their capacity to mitigate resistance [20].

**Figure 3:**
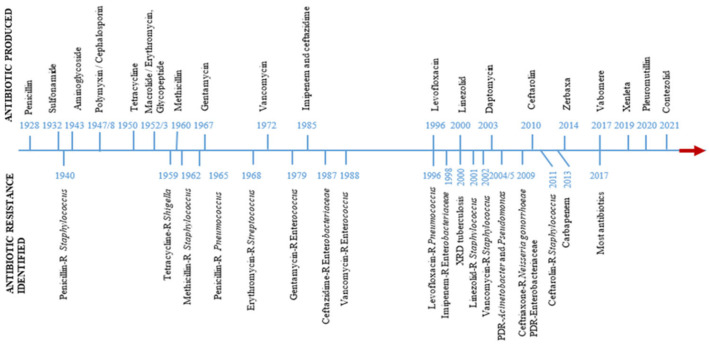
Timeline showing the discovery of antibiotics and the rise of antibiotic resistance [21].

The historical progression of antibiotic discovery and resistance development is shown in Figure 3.

#### 2.3.2. AMR in Agricultural Runoff and Manure

Agricultural runoff represents a major conduit for the environmental dissemination of AMR, bridging animal agriculture with aquatic and terrestrial ecosystems. In livestock and poultry production, antibiotics are frequently administered for therapeutic, prophylactic, and growth-promoting purposes. However, up to 90% of the administered antibiotics are excreted unmetabolized in urine and feces, rendering animal manure a rich source of antibiotic residues, ARB, and ARGs [22] especially in middle/high-income countries.

When such manure is applied to agricultural fields or stored in open lagoons, rainfall and irrigation can mobilize these contaminants via surface runoff and subsurface leaching, facilitating their entry into rivers, lakes, and groundwater systems. These environmental compartments then serve as reservoirs and mixing grounds for resistance determinants. Moreover, sub-inhibitory concentrations of antibiotics in runoff exert selective pressure on environmental microbiota, accelerating HGT and fostering the emergence of multidrug-resistant organisms [23].

Soil microbial communities exposed to repeated manure applications may develop stable reservoirs of ARGs, which can be mobilized by human pathogens through mobile genetic elements such as plasmids, transposons, and integrons [24]. These pathogens may enter the human food chain through crops irrigated with contaminated water or fertilized with untreated manure.

Additionally, co-selection pressure from heavy metals and biocides commonly present in agroecosystems owing to pesticide use and mineral supplementation can further enhance the persistence and spread of AMR through shared resistance mechanisms [25].

From a One-Health perspective, mitigating AMR in agricultural runoff necessitates a multi-sectorial approach encompassing veterinary stewardship, optimized manure management (e.g., composting, anaerobic digestion), eco-engineered buffers (e.g., constructed wetlands), and monitoring of ARGs in agro-environments. Such integrated interventions are critical in interrupting AMR transmission across the human–animal-environment interface.

Humans and animals are exposed through contaminated crops, animal products, and water supplies. Resistant pathogens originating in farms or industrial effluents can thus cycle back into clinical and veterinary settings.

#### 2.3.3. AMR in Municipal Sewage

Municipal sewage systems collect waste from households, healthcare facilities, and industries, making them a critical nexus in the transmission of AMR across human, environmental, and animal health domains. Human excreta contain unmetabolized antibiotics, ARB, and ARGs, especially in communities with high antibiotic consumption or poor pharmaceutical stewardship [26].

WWTPs, although designed to reduce microbial loads and organic matter, are not fully effective in removing antibiotics or resistance determinants. Studies have detected ARGs in treated effluents, sludge, and biosolids, indicating that WWTPs may act as reservoirs and amplification points for AMR. Effluents discharged into rivers, lakes, or used for irrigation can disseminate resistance genes into natural ecosystems and agricultural soils, where environmental bacteria or crop-associated microbiota [27] may take them up.

Furthermore, the co-existence of diverse microbial communities in WWTPs fosters HGT through mobile genetic elements such as plasmids and integrons, facilitating the evolution of multidrug-resistant organisms. These may ultimately re-enter the human population via drinking water, food chains, or direct environmental contact, completing the One-Health cycle.

Effective mitigation requires advanced treatment technologies (e.g., membrane filtration, UV, ozonation), routine monitoring of ARGs in effluents, and coordinated action across public health, environmental management, and wastewater engineering sectors.

#### 2.3.4. AMR in WWTP Discharges

WWTPs are essential for mitigating pollution from domestic, hospital, and industrial effluents. WWTPs are increasingly identified as critical hotspots for the emergence and dissemination of AMR environments where AMR determinants are detected at elevated levels, due to likely linked to the incomplete removal of antibiotic residues, ARB, and ARGs during treatment processes [28].

Antibiotics enter WWTPs via human and animal excreta, hospital wastewater, and pharmaceutical industry discharges. Conventional treatments such as primary sedimentation, biological treatment (e.g., activated sludge), and secondary clarification are effective in reducing organic pollutants and pathogens but often fail to fully eliminate antibiotics and ARGs [26].

Studies have shown that antibiotics like sulfonamides, fluoroquinolones, tetracyclines, and β-lactams persist throughout the treatment chain, sometimes even increasing in relative abundance due to selective microbial pressure [29]. Furthermore, ARGs conferring resistance to multiple drug classes have been detected in both treated effluents and sewage sludge [30], which, when applied to agricultural land as biosolids, contributes to environmental ARG reservoirs.

The high microbial density and presence of mobile genetic elements (e.g., plasmids, integrons, transposons) in WWTPs create ideal conditions for HGT, facilitating ARG exchange between environmental microbes and potential human pathogens [26,31].

Effluent discharge into rivers, lakes, and coastal waters becomes a chronic source of AMR contamination, altering microbial ecology and enabling the spread of ARB has been associated with higher detection of AMR determinants, which may influence microbial ecology and potentially facilitate the spread of ARB in aquatic organisms [32]. Additionally, the reuse of treated wastewater for irrigation can transfer ARGs to agricultural soils and crops, increasing the risk of human and animal exposure [24].

To address these risks, advanced treatment technologies such as membrane filtration (ultrafiltration, nanofiltration) for physical removal of bacteria and ARGs, advanced oxidation processes (e.g., ozonation, UV, Fenton reactions) for antibiotic degradation, and constructed wetlands or biologically activated carbon systems for tertiary contaminant removal are being explored and implemented [26].

Integrating these technologies with routine monitoring of AMR determinants is essential to reduce environmental dissemination and safeguard public health within a One-Health framework.

Humans may be exposed via contaminated drinking water, seafood, and irrigated crops, while animals encounter these contaminants through water sources and fodder. WWTPs thus represent amplification points where resistance traits are transferred between microbial communities before re-entering human and animal populations.

#### 2.3.5. AMR in Soil and Water Systems

Soil and water systems act as major environmental reservoirs and conduits for AMR. These ecosystems receive continuous inputs of antibiotic residues, ARB, and ARGs from diverse sources such as agricultural runoff, animal manure, municipal sewage, and pharmaceutical industry effluents [33].

In soil, repeated application of contaminated manure or biosolids foster the accumulation of ARGs. These genes can persist for long periods and may even be taken up by native microbes through HGT, particularly under the selective pressure of antibiotics or co-selective agents like heavy metals and pesticides. This creates a complex microbial network capable of transmitting resistance to clinically important pathogens [34].

Water bodies, including rivers, lakes, groundwater, and irrigation canals, act as transport systems for AMR. Antibiotics and ARGs carried in surface runoff or treated/untreated wastewater can alter aquatic microbial communities, reduce biodiversity, and enable the proliferation of resistant strains [27]. Sediments further act as long-term sinks for ARGs, supporting microbial gene exchange over time.

From a One-Health standpoint, AMR in soil and water systems links environmental contamination directly to risks for human and animal health through exposure via food, drinking water, and recreational use. Addressing this issue requires integrated efforts in land use planning, sustainable agriculture, wastewater treatment upgrades, and environmental surveillance.

A study conducted in the soil and the rhizosphere of plants grown in mercury-contaminated sites highlighted that mercury contamination indirectly contributes to the spread of AMR. Mercury promotes the co-selection of ARGs that confer resistance to different antibiotics in *Bacillus* spp. Among the isolates tested, 72% of *Bacillus* spp. exhibited resistance to two or more commonly used antibiotics, indicating a high prevalence of multidrug resistance (MDR). Additionally, 38 isolates demonstrated resistance to cephalosporins, a critical class of β-lactam antibiotics. These findings suggest a potential co-selection between heavy metal tolerance and antibiotic resistance traits in environmental *Bacillus* populations [14,35].

#### 2.3.6. AMR in Wastewater Samples

Wastewater sampling has emerged as a powerful tool for AMR surveillance and environmental monitoring. It offers critical insights into the dissemination and evolution of resistance across human, animal, and environmental interfaces, aligning closely with the One-Health framework.

Wastewater contains a complex mixture of antibiotic residues, ARB, and ARGs excreted from humans, animals, and discharged from pharmaceutical or hospital sources. These compounds can persist through conventional treatment processes and enter aquatic ecosystems, promoting the spread and enrichment of resistance in natural microbial communities [13,36].

Monitoring wastewater enables early detection of emerging resistance trends at the population level, often before clinical detection. It also reflects AMR burden from both symptomatic and asymptomatic individuals, animals, and industrial sources, making it a non-invasive, cost-effective surveillance tool [37].

By capturing resistance determinants from urban sewage, agricultural runoff, and industrial discharges, wastewater integrates signals from all three One-Health sectors. This makes it a critical medium for comprehensive AMR surveillance, especially in regions lacking robust clinical monitoring infrastructure.

Wastewater effluents, particularly when untreated or poorly treated, contribute to the contamination of surface waters, soils, and irrigation systems. ARGs and ARB in these waters can be taken up by plants, enter aquaculture systems, or persist in the environment, enabling HGT and the proliferation of multidrug-resistant pathogens [36,38]. This poses direct risks to human and animal health through food, water, and environmental exposure.

Incorporating wastewater surveillance into AMR monitoring frameworks exemplifies the One-Health approach in action. It bridges human, animal, and environmental health domains, offering a scalable, real-time tool to track and mitigate AMR across sectors.

#### 2.3.7. AMR in Marine Sediment

Marine sediments are emerging as critical reservoirs of AMR in the environment, representing a complex interface between anthropogenic pollution and natural ecosystems. Within the One-Health framework, marine sediments reflect the downstream accumulation of antibiotic residues, ARB, and ARGs originating from human activities, including municipal sewage discharges, aquaculture effluents, industrial runoff, and hospital wastewater [39].

Antibiotics and resistance genes introduced into coastal and estuarine waters settle and persist in sediments due to sorption to particulate matter. These conditions promote selective pressure on native microbial communities, facilitating HGT and the proliferation of multidrug-resistant organisms [40]. Sediment microbiota often carry mobile genetic elements such as integrons and plasmids, which can harbor clinically relevant resistance genes including those conferring resistance to β-lactams, sulfonamides, and tetracyclines [41].

The use of antibiotics in aquaculture further compounds this issue, as medicated feed and unregulated antibiotic application result in the direct deposition of residues and ARB into marine sediments [42]. These sediments serve as long-term reservoirs, posing a risk of reintroducing resistant bacteria into marine food webs through benthic organisms and potentially reaching humans via seafood consumption.

From a One-Health standpoint, surveillance of AMR in marine sediments is essential to track the environmental dissemination of resistance and its potential feedback to human and animal populations. Integrated monitoring programs, source tracking of ARGs, and regulation of coastal pollution are vital for disrupting the environmental persistence of AMR and protecting marine and public health.

#### 2.3.8. AMR in Aerosols

AMR in aerosols represents a critical yet underexplored vector of environmental and public health concern within the One-Health framework, which integrates human, animal, and environmental health. Aerosols are fine solid or liquid particles suspended in air, capable of transporting microorganisms over varying distances. Recent studies indicate that airborne dissemination of ARB and ARGs can occur through aerosols originating from animal farms, WWTPSs, healthcare settings, and even urban environments. For instance, poultry and swine facilities have been identified as major reservoirs of airborne AMR due to the frequent use of antibiotics for growth promotion and disease prevention.

Aerosolized particles from these environments can contain viable ARB such as *Escherichia coli*, *Staphylococcus aureus*, and *Klebsiella pneumoniae*, often harboring resistance determinants like blaCTX-M, mecA, and tetM [43]. These bioaerosols, once inhaled or deposited on surfaces, may facilitate HGT in new hosts, further propagating resistance.

The aerosol pathway exemplifies the One-Health complexity of AMR dissemination. In agricultural settings, inhalation of contaminated dust by farm workers poses direct risks to human health [44]. Simultaneously, airborne particles can settle into soil and water systems, influencing microbial communities and potentially entering the food chain. Moreover, migratory birds and wind currents can facilitate the transboundary movement of aerosolized AMR, further complicating containment strategies [10]. Healthcare environments are also increasingly implicated in aerosol-mediated AMR transmission, especially in poorly ventilated settings, where aerosol-generating medical procedures may disperse resistant strains among hospital patients and staff [45].

To address the airborne dimension of AMR, environmental surveillance must be integrated into One-Health monitoring systems. This includes air sampling in high-risk areas such as intensive animal production units, hospitals, and urban wastewater sites. Advanced molecular techniques like metagenomic sequencing and quantitative PCR enable the detection of ARGs in aerosol samples, providing crucial data for risk assessment [46].

Mitigation strategies should include improved ventilation and dust control in animal and healthcare settings, rational antibiotic use across sectors, use of personal protective equipment (PPE) for workers, and the development of interdisciplinary policies within the One-Health framework.

The potential for aerosol transmission of AMR underscores the need for a multisectoral and transdisciplinary approach. As airborne dissemination bridges environmental, human, and animal interfaces, it exemplifies a critical but often underappreciated component of AMR dynamics. Future research should focus on quantifying exposure risks, understanding aerosol behavior in diverse settings, and establishing standardized protocols for monitoring airborne AMR under the One-Health paradigm.

Aerosol transmission is thus a major route for respiratory pathogen spread. Detection methods include culture, which confirms viability but is slow; qPCR, which is rapid but detects both viable and non-viable nucleic acids; and metagenomics, which profiles microbial diversity without indicating infectivity [47,48]. Viral concentrations in aerosols typically range from 10 to 10,000 copies/m^3^, mainly in particles under 5 µm that can penetrate deep into the lungs [49,50]. Risk assessment frameworks classify environments by aerosol load and ventilation, recommending basic controls for low-risk areas, enhanced ventilation and filtration for moderate risk, and strict respiratory protection and engineering controls for high-risk settings. This tiered approach guides effective aerosol transmission mitigation.

### 2.4. Aerosols and Marine Sediments as Overlooked AMR Pathways

Aerosols and marine sediments are becoming recognized as important reservoirs and transmission routes for AMR. Aerosolized dust can carry bacteria like *E. coli*, *Staphylococcus aureus*, and *Klebsiella pneumoniae* with resistant gene such as blaCTX-M and tetM from sites like farms and wastewater treatment plants, potentially exposing humans and wildlife. Marine sediments also accumulate resistant microbes and ARGs from wastewater, which can be remobilized and spread through coastal environments [51].

Airborne particles, soil, water, and marine sediments serve as reservoirs for antibiotic residues and resistance genes, which can spread through environmental contamination and HGT. These environmental reservoirs pose risks to humans and ecosystems by contributing to the global burden of AMR, potentially re-entering the food chain through seafood and agricultural products. To mitigate these risks, it is necessary to implement stronger surveillance and intervention strategies in aerosol and marine environments to better understand AMR dynamics and reduce its spread [32].

#### AMR in Wildlife

Wild animals like birds, rodents, and bats play an unexpected but important role in the spread of AMR. These animals often are exposed to contaminated environments, such as polluted water, agricultural runoff, or waste from farms and cities. When exposed, they can pick up ARB and ARGs and may even carry them over long distances, especially in case of examples such as migratory birds [52].

Wildlife does not just carry the bacteria they can also help them evolve. In their gut or in shared environments, resistance genes can be transferred between different microbes. This gene swapping, known as HGT, can lead to the development of new resistant strains, including ones that are harmful to humans [53].

Studies have found superbugs like ESBL-producing *E. coli* and MRSA in wild birds, foxes, and other animals even in remote areas. This shows that AMR is not just a problem created by hospitals or farms, it is spreading across the natural world too [54].

From a One-Health perspective, which connects human, animal, and environmental health this highlights why we need better monitoring of AMR in wildlife, reduced antibiotic use in agriculture, and establish cleaner environments to break the chain of resistance transmission.

Humans and domestic animals may be exposed through food webs, direct contact with contaminated water or wildlife, and environmental reservoirs that act as gene pools for emerging resistant pathogens.

### 2.5. AMR and Its One Health Implication on Livestock

AMR in livestock is a growing global health concern that directly impacts human health and environmental ecosystems. The One Health framework underscores the interconnectedness of human, animal, and environmental health, and AMR is a prime example of this interdependence. This section explores the major livestock sectors cattle, camels, poultry, and fish with a focus on antimicrobial misuse, environmental reservoirs, and food chain transmission. The key challenge is to reduce antimicrobial misuse in livestock while addressing the environmental spread of resistant bacteria and genes [55].

#### 2.5.1. Antimicrobial Use in Livestock and Its Environmental Impacts: A One-Health Perspective

The intensive use of antibiotics in livestock farming, primarily for therapeutic, prophylactic, and growth-promoting purposes, has led to significant environmental consequences. It is estimated that approximately 30–90% of administered antibiotics are excreted unchanged in urine and feces, which, when stored or applied to agricultural land as fertilizer, creates a major reservoir of antibiotic residues, ARB, and ARGs [56]. These residues, when introduced into the environment through manure storage or land application, can contaminate soil and water via runoff or leaching, promoting the persistence and proliferation of ARB and ARGs, which can be transferred across species, including to humans [57].

Animal waste, often stored in lagoons or piles, becomes a selective environment under the pressure of antibiotic residues, facilitating the HGT of resistance genes. Sub-inhibitory concentrations of antibiotics may not directly kill bacteria but can still select for resistant strains, accelerating mutation and recombination [12]. Soil microflora can serve as long-term reservoirs of ARGs, which can be taken up by pathogens, creating a pathway from the environment to humans via crops or water [58]. Moreover, agricultural chemicals such as heavy metals and biocides, which share resistance mechanisms with antibiotics, can exacerbate the persistence of AMR in animal waste. These multi-resistance genes can be selected even in the absence of antibiotics, complicating control efforts [25].

The One-Health approach underscores the interconnectedness of human, animal, and environmental health, illustrating the serious implications of AMR in livestock production. Resistant bacteria can be transmitted to humans through direct contact with animals, consumption of contaminated animal products such as meat or milk, and environmental pathways like water contaminated with animal waste. Manure, when used as fertilizer, can introduce resistance genes into the soil and water, perpetuating the environmental reservoir of AMR [59,60]. In cattle farming, antimicrobials are often used to manage diseases like mastitis, respiratory infections, and gastrointestinal disorders. However, overuse and misuse of drugs like tetracyclines, penicillins, and macrolides can promote the emergence of resistant bacteria such as *E. coli*, *Salmonella*, and *Staphylococcus aureus*. These resistant strains can then be transmitted to humans, posing significant public health risks [61,62]. Similarly, poultry farming faces similar challenges related to AMR, with the misuse of antibiotics leading to resistant strains that can spread to humans through contaminated food or environmental exposure [63].

Mitigating AMR in livestock requires integrated strategies. Prudent antibiotic use in veterinary care must be accompanied by the promotion of alternatives, such as probiotics, prebiotics, and phytochemicals, which can help reduce the reliance on antibiotics [64,65,66,67,68,69,70,71,72]. Manure treatment methods like composting or anaerobic digestion can significantly reduce the ARGs in waste, while runoff containment measures can prevent environmental contamination and limit the spread of ARB and ARGs. Enhanced farm hygiene, biosecurity measures, and surveillance systems are also crucial in monitoring and controlling the spread of resistance. International organizations such as the Food and Drug Administration (FDA) and the WOAH have been pushing for tighter regulations on antimicrobial use in livestock, particularly non-therapeutic uses. Stricter guidelines are necessary to reduce over-reliance on antibiotics and curb the emergence of resistant strains. Coordination across sectors and countries is crucial to harmonize policies, share data, and develop alternatives to antibiotics, such as vaccines and improved farming practices [62,73].

The One-Health perspective reinforces the need for cross-sector collaboration to tackle AMR. This includes promoting antimicrobial stewardship, enhancing surveillance systems, and fostering international cooperation to protect public health, safeguard food security, and protect the environment. Without such coordinated efforts, the spread of AMR from livestock to humans and the environment could have devastating consequences on global health.

#### 2.5.2. AMR and Its One-Health Implication on Cow and Camel Milk

Milk, a vital component of human nutrition, particularly in arid and semi-arid regions, is increasingly recognized as a potential vehicle for the transmission of AMR to humans. Cow and camel milk, which are widely consumed for their nutritional and therapeutic benefits, can act as reservoirs for ARB and ARGs. These resistance elements may be transferred to humans through the consumption of raw or improperly processed milk, especially when sourced from animals treated with antibiotics or raised in unhygienic conditions. This presents a significant public health concern, highlighting the interconnectedness of human, animal, and environmental health as emphasized in the One-Health framework [74,75].

#### 2.5.3. AMR in Cow

Antibiotic residues, particularly from tetracyclines, sulfonamides, and quinolones, are commonly found in cow milk, especially from animals affected by mastitis. Multidrug-resistant (MDR) *Escherichia coli* and *Salmonella* strains are frequently isolated from raw milk [63,76]. Studies from Bangladesh found that approximately 29.8% of raw cow milk samples contained *E. coli*, most of which were resistant to commonly used antibiotics like ampicillin and cephalosporins. Similarly, in Egypt, 70% of raw milk samples tested positive for MDR *E. coli* and *Staphylococcus aureus*. These bacteria carry resistance genes like blaTEM, which confers resistance to beta-lactam antibiotics, and blaCTX-M, which confers resistance to extended-spectrum beta-lactams. The consumption of raw or improperly processed milk can transmit these resistant strains to humans, presenting a significant public health concern [77,78].

#### 2.5.4. AMR in Camel Milk

Camel milk, particularly in regions like the Middle East and Africa, is often consumed raw for its nutritional and medicinal benefits. However, camel milk can also harbor antibiotic-resistant bacteria, including *Klebsiella pneumoniae*, *Escherichia coli*, and *Staphylococcus aureus*. Research from Egypt showed that 30% of camel milk samples from animals with mastitis contained *S. aureus*, with 50% of these strains resistant to multiple antibiotics. A study in Kenya found *Clostridium perfringens* in 19% of camel milk samples, with high resistance to antibiotics such as ampicillin and tetracycline. The consumption of raw camel milk or its improper processing can expose humans to multidrug-resistant bacteria, leading to potential health risks, particularly in communities where raw milk is consumed frequently [79,80,81,82].

#### 2.5.5. AMR in Poultry

AMR in poultry is a growing concern, with bacteria such as Escherichia coli, Salmonella, and Campylobacter frequently harboring resistance to multiple antibiotics. These resistant bacteria can persist in poultry feces, which can then contaminate the environment through runoff or poor waste management practices. Resistance genes from poultry can also spread through the food chain, particularly when meat is undercooked or cross-contaminated during handling. In the Netherlands, studies found that poultry workers had a higher carriage rate of multidrug-resistant E. coli compared to the general population. The widespread use of antibiotics like tetracyclines, fluoroquinolones, and macrolides in poultry farming, often without adequate veterinary oversight, has facilitated the spread of resistance. The contamination of soil and water with resistant bacteria from poultry manure further exacerbates the problem [83,84,85,86].

#### 2.5.6. AMR in Fish

Fish farming is an essential source of protein globally, but the excessive and inappropriate use of antibiotics in aquaculture contributes significantly to AMR. Resistant bacteria in fish farms can spread to the aquatic environment and eventually enter the human food chain through consumption or occupational exposure. In aquaculture, the discharge of untreated or poorly treated effluents can introduce antibiotic residues and resistant bacteria into natural water bodies, amplifying the spread of resistance genes. Studies in Thailand have linked antibiotic resistance in fish farming to the discharge of untreated effluents into water systems. As with other forms of livestock farming, the environmental reservoirs created by fish farming play a key role in the persistence and spread of AMR [39,,87,88].

The One-Health framework emphasizes that the health of humans, animals, and the environment is interconnected. The transmission of AMR from livestock to humans occurs through multiple routes, including direct contact with animals, consumption of contaminated animal products, and exposure to environmental reservoirs of resistant bacteria. These resistant bacteria can then spread within human communities, exacerbating public health risks and complicating treatment options for common infections [62,73].

Environmental reservoirs of AMR, particularly in water and soil, are crucial to understanding how resistance spreads. In all livestock sectors, manure acts as a significant source of ARGs, which can be transferred to the environment and, in turn, back to animals and humans through various pathways. Addressing AMR in livestock, therefore, requires a comprehensive approach that includes monitoring and controlling environmental contamination, improving animal health practices, and ensuring responsible antibiotic use [60,62,89].

Several strategies are essential for mitigating AMR in livestock. First, prudent antibiotic use is crucial. Antibiotics should only be administered when necessary, with a focus on treating specific infections rather than for prophylaxis or growth promotion. Veterinary practices should emphasize responsible prescription, including adherence to withdrawal periods, to prevent antibiotic residues in food products [64,72].

Manure management plays a key role in preventing environmental contamination. Techniques such as composting, anaerobic digestion, and runoff containment can significantly reduce the spread of ARGs from manure. Additionally, alternative practices like using probiotics, prebiotics, and vaccines can reduce the reliance on antibiotics, promoting healthier livestock while minimizing the risk of resistance [64,72,77].

Public education and training for farmers, veterinarians, and food safety workers are critical. In regions like Punjab, India, educational programs have successfully improved understanding of AMR risks, leading to better antibiotic practices and enhanced farm hygiene. These efforts help foster a culture of responsible antibiotic use and environmental stewardship.

### 2.6. A One-Health Prospective of AMR in Humans

Antibiotics have played a transformative role in clinical medicine since their discovery, dramatically reducing mortality and morbidity from bacterial infections such as pneumonia, sepsis, meningitis, and tuberculosis. Their therapeutic application extends beyond active treatment, providing prophylactic protection during surgeries, chemotherapy, and organ transplantation. This foundational role in infection control supports a wide range of medical procedures that would otherwise carry unacceptable risks.

In routine healthcare, antibiotics are indispensable for managing both common infections such as urinary tract infections and streptococcal pharyngitis and life-threatening conditions like bacterial meningitis and septicemia [90]. They also facilitate complex surgeries and immunosuppressive therapies by preventing opportunistic infections [91]. The effectiveness of antibiotics in these contexts depends on appropriate use accurate targeting, correct dosing, and complete treatment courses. When used judiciously, antibiotics are powerful tools for saving lives and improving health outcomes.

AMR arises when microorganisms evolve mechanisms to resist the effects of antimicrobial agents. This resistance emerges primarily through spontaneous genetic mutations and HGT via plasmids, transposons, integrons, or through conjugation, transformation, and transduction [85]. These processes enable the rapid spread of resistance traits among microbial populations, especially under selective pressure from excessive antimicrobial exposure in human, veterinary, and agricultural settings. At the molecular level, resistance mechanisms include enzymatic inactivation (such as β-lactamases), target site modifications (like methylation of 23S rRNA); efflux pumps that expel drugs from bacterial cells, and reduced membrane permeability that prevents drug entry [86]. These adaptations collectively undermine antimicrobial efficacy and contribute to the survival and dominance of resistant strains.

From a clinical perspective, AMR leads to treatment failures in infections that were once readily manageable. Resistant pathogens, such as multi-drug-resistant *Mycobacterium tuberculosis* and CRE, are associated with increased mortality, prolonged hospital stays, and higher treatment costs [1]. First-line antibiotics often become ineffective, forcing clinicians to rely on second- or third-line agents, which are typically more toxic, less effective, and more expensive.

Resistant organisms like *Escherichia coli* resistant to fluoroquinolones and third-generation cephalosporins [87] increasingly cause infections such as complicated urinary tract infections, pneumonia, sepsis, and sexually transmitted infections []. Several interrelated factors contribute to the rise of AMR including overuse and misuse of antibiotics in humans and animals, inappropriate prescribing especially for viral infections patient non-compliance with treatment regimens, and empirical use of broad-spectrum antibiotics in settings without rapid diagnostic tools. Transmission of resistant bacteria occurs not only in healthcare environments, where hospital-acquired infections and intensive antibiotic use promote spread, but also in community settings through poor hygiene, contaminated water, and inadequate sanitation [87]. Consequently, AMR poses a complex challenge that extends far beyond clinical settings.

AMR has diverse genetic mechanisms including enzymatic inactivation by enzymes like β-lactamases, efflux pumps that expel antibiotics, and target site modification altering antibiotic binding. Resistance genes are spread via mobile genetic elements like plasmids, conferring resistance across human, animal, and environmental contexts, highlighting HGT’s role in dissemination under selective pressure from antibiotic residues [92].

#### AMR in Retrospective Hospital Infection Data Analysis

Retrospective hospital infection data analysis plays a pivotal role in understanding the epidemiology of AMR within the clinical setting, providing crucial insight into the human health dimension of the One-Health framework. Hospitals act as critical surveillance points for identifying trends in healthcare-associated infections (HAIs) and the emergence and dissemination of antibiotic-resistant organisms (AROs), such as MRSA, CRE, and multidrug-resistant *Acinetobacter baumannii* [14].

Retrospective analysis involves mining historical patient records, laboratory data, antibiotic prescriptions, and infection control logs to evaluate infection patterns, risk factors, and outcomes [93]. These data help quantify the burden of AMR, inform empirical treatment guidelines, and assess the effectiveness of antimicrobial stewardship interventions [94].

The most commonly reported AMR prevalences in clinical settings were among the Gram-negative isolates, including *E. coli*, *K. pneumoniae*, *P. aeruginosa*, and *Acinetobacter baumannii*.

Furthermore, linking hospital AMR data with community and environmental sources of resistance (e.g., wastewater, livestock exposure, or travel history) facilitates a holistic One-Health risk assessment. For instance, retrospective evidence has shown correlations between hospital-acquired infections and resistance profiles observed in municipal sewage or animal production settings, suggesting a bidirectional flow of resistance determinants across sectors [13,22].

However, such analyses are often limited by incomplete records, lack of standardized surveillance protocols, and difficulties in linking hospital isolates with environmental or veterinary AMR data. Nonetheless, improving data integration across the One-Health spectrum of human, animal, and environmental health is essential for effective AMR containment.

A comparative overview of AMR prevalence, drivers, and interventions across human, animal, and environmental sectors is summarized in Table 1.

**Table 1:** Comparative Overview of AMR Across Human, Animal, and Environmental Sectors within the One-Health Framework.

Sector/Source	Sentinel ARGs/Pathogens	Dominant Drivers	Transmission Pathways	Priority Interventions	Critical Knowledge Gaps
Human ^1^	MRSA, CRE (*K. pneumoniae*, *E. coli*), MDR *A. baumannii*; genes:blaCTX-M, mecA ^1^	Inappropriate prescribing; empirical broad-spectrum use; poor infection control	Person-to-person, healthcare settings, contaminated food and water	Stewardship, rapid diagnostics, infection control, integrated surveillance	Limited integration with environmental/veterinary data; under-reporting in LMICs
Livestock ^2^	ESBL-*E. coli*, *Salmonella*, *S. aureus*, MRSA, *E. faecium*; genes: blaCTX-M, blaTEM, qnrS, tetA, mcr-1, tetK, ermB, vanA)^2^	Routine antibiotic use for prophylaxis, therapy, growth promotion, Antibiotic residues in milk; mastitis treatment; raw milk consumption	Direct contact, contaminated meat and milk, environmental contamination	Restricting antibiotic use, implementing biosecurity measures, promoting vaccination, surveillance	Effectiveness of stewardship in smallholder systems; farm-to-fork tracking
Poultry ^3^	*E. coli*, *Salmonella*, *Campylobacter*; genes: qn*r*, te(), bla_CTX-*M*, mcr-1^2^	High antimicrobial use for growth promotion & prevention; poor hygiene	Direct contact, contaminated eggs and meat, environmental sources	Farmer education, improved hygiene and biosecurity, restricted antibiotic use, surveillance	Limited LMIC data; few intervention trials; dose–response unknown
Environment ^4^	*E.coli*, *Enterobacter* spp.	Manure runoff, sewage, wastewater treatment plant (WWTP) effluents	Water, air, soil, crops, food chain	Advanced wastewater treatment, monitoring of effluent discharge, environmental surveillance	Standardized environmental protocols lacking; causality links unclear; few long-term resistance datasets
Aquaculture ^5^	*Vibrio* spp., *Aeromonas* spp.; genes: flo*R*, su*l*1, te(), mcr-1	Unregulated antibiotic use, poor water quality	Contaminated water, seafood consumption, environmental sources	Probiotics, vaccines, effluent regulation, improved aquaculture practices	Lack of global regulation; poor effluent tracking; weak LMIC data
Wildlife ^6^	ESBL-*E. coli*, *MRSA*; genes: bl_CTX-*M*, bl(), va()	Environmental exposure (runoff, waste); migration spread	Migratory spread, environmental reservoirs, contact with contaminated sources	Wildlife surveillance, habitat protection, monitoring of migratory patterns	Lack of global monitoring; unclear transmission back to humans
Wastewater/WWTPs ^7^	*E. coli*, *Enterobacter* spp.; genes: blaCTX-*M*, sul1, tetA)	Hospital, pharmaceutical, municipal effluents; incomplete treatment	Treated and untreated effluent entering rivers, lakes, irrigation water, sludge reuse	Advanced treatment (UV, ozonation, membranes), ARG monitoring, industrial discharge regulation	Standardized protocols lacking; unclear causality; limited time-series resistance data
Aerosols ^8^	*E. coli* , *K. pneumoniae*, *S. aureus*; genes: *blaCTX-M*, *mec*(*A*), *tet*(*M*)	Dust from farms, WWTPs, hospitals; poor ventilation	Inhalation of contaminated dust and bioaerosols; occupational exposure	Air sampling, PPE, ventilation upgrades, exposure risk studies	No standardized protocols; dose–response missing

(Source: ^1^ [87,90], ^2^ [55,61], ^3^ [59,80], ^4^ [33], ^5^ [36,37], ^6^ [55,58], ^7^ [24,93], ^8^ [42,43]).

### 2.7. Public Health and Economic Impacts

Drug-resistant pathogens are responsible for a wide spectrum of complications in both community and hospital settings, including urinary tract infections, sepsis, pneumonia, wound infections, and foodborne diseases [2]. The failure of first-line antibiotics forces reliance on second- or third-line agents, which are often less effective, more toxic, and significantly more expensive. These results in longer hospitalizations, higher treatment costs, increased surgical risks, and greater morbidity and mortality [2,93].

In LMICs, limited resources for infection prevention, weak surveillance, and constrained access to second-line antimicrobials result in higher case fatality rates and greater transmission of resistant pathogens [13,88]. The World Bank warns that by 2050, AMR could push an additional 24 million people into extreme poverty, the majority of whom will be in LMICs [3]. These inequities underscore the urgent need for targeted investments in diagnostics, surveillance infrastructure, and water, sanitation, and hygiene (WASH) systems in resource-limited settings [13,73].

From an economic perspective, AMR leads to increased outpatient visits, expensive diagnostics, and escalated therapeutic interventions. The financial impact is significant: the World Bank warns that by 2050, AMR could cause a 1.1–3.8% reduction in global GDP and push an additional 24 million people into extreme poverty [3]. LMICs will have to contend with the worst of this impact due to weaker surveillance systems, limited access to new antimicrobials, and underfunded healthcare systems [13].

AMR in livestock, poultry, and aquaculture leads to increased mortality, reduced productivity, and higher veterinary costs, while also restricting trade and threatening global food security [46,94]. Zoonotic transmission of resistant pathogens through animal products and environmental exposure further intensifies the public health threat [91].

The economic burden in high-prevalence regions is amplified by the need for ICU-level care, use of last-resort antimicrobials like carbapenems and colistin, and implementation of robust infection control measures. Furthermore, environmental contamination from pharmaceutical and hospital waste that contributes to the spread of resistance through water systems, aquaculture, and the food chain [95] requires careful attention.

Without urgent and coordinated interventions, the cumulative public health and economic burden of AMR is expected to rise exponentially, threatening decades of progress in global health, sustainable development, and poverty reduction.

### 2.8. Socioeconomic Disparities and AMR in LMICs

Socioeconomic disparities exacerbate AMR in LMICs by restricting access to quality healthcare, fueling self-medication and misuse of antibiotics, and promoting poor infection control [96]. These disparities also heighten the impact of AMR, disproportionately affecting vulnerable populations with greater GDP and productivity losses, and creating a cycle where AMR further entrenches socioeconomic inequalities. Addressing AMR requires integrated strategies focusing on improved healthcare access, strong regulations, public education, and community engagement to tackle the underlying socioeconomic drivers [97].

### 2.9. Future Outlook and Strategies for Containment

The future of AMR largely depends on how well we can work together across different sectors encompassing human health, animal health, and the environment under what is called the One-Health approach. By connecting these fields, we can better stop resistance from developing and spreading through various pathways [73]. One of the most important steps is improving antimicrobial stewardship that means strengthening antimicrobial stewardship to ensure rational prescribing practices in both human and veterinary medicine. Without this, the development and spread of resistance in the environment will only worsen.

Another key area that requires urgent attention is building strong surveillance and monitoring systems that track AMR not only in people but also in farms and the environment. This includes keeping an eye on wastewater and marine environments where resistant bacteria can evolve and accumulate. On the environmental front, adopting advanced wastewater treatment technologies such as ozonation, UV treatment, and membrane filtration at hospitals and industrial zones can significantly reduce the release of antibiotics and resistance genes into natural ecosystems.

Managing manure and agricultural runoff is also critical. Methods like composting, anaerobic digestion, and establishing buffer zones can help prevent ARGs from spreading through soil and water. In livestock and aquaculture, encouraging the use of vaccines, probiotics, and plant-based alternatives to antibiotics offers promising ways to reduce reliance on antimicrobials while maintaining animal health [61].

Educating people is equally vital, raising public awareness and providing targeted training for farmers, healthcare workers, and communities can foster better behaviors around antibiotic use and hygiene [73]. Finally, all these efforts need to be supported by strong policy frameworks and international collaboration. Agencies like the WHO, FAO, and WOAH play key roles in setting standards and facilitating global knowledge sharing.

Future research should prioritize meta-analytic syntheses of ARG prevalence across sectors. Pooled analyses would allow more robust estimates of resistance gene burdens, identify high-risk reservoirs, and clarify regional variations. Such quantitative approaches would strengthen the evidence base for prioritizing interventions under the One Health framework

## 3. Discussion

This review highlights the complex and interconnected drivers of AMR across human, animal, and environmental domains. Consistent with prior literature, pathways such as HGT are recognized as central to resistance dissemination. However, the causal links between environmental reservoirs, wildlife, and human clinical outcomes remain insufficiently substantiated. For example, studies have detected resistant *E. coli* and *Staphylococcus aureus* in wild birds and mammals, but the extent to which wildlife populations amplify or transmit resistance back to humans is still poorly quantified. Rather than assuming a uniform role, wildlife should be considered as potential sentinels or reservoirs whose risks to human health require further systematic study [98].

Similarly, the environmental sector, particularly wastewater treatment plants, pharmaceutical effluents, and agricultural runoff, is often described as a major AMR hotspot. While evidence supports the persistence of antimicrobial residues and resistance genes in these systems, attributing direct causal links to human infections is challenging. Statements about environmental drivers must therefore be contextualized as associations rather than definitive sources, pending more robust epidemiological and genomic tracing studies [54]. Nonetheless, isolate-based investigations have provided supporting evidence of resistant pathogens circulating at this interface, such as multidrug-resistant *Klebsiella pneumoniae* from clinical and environmental sources [99] and resistant *E. coli* and *Staphylococcus aureus* recovered from dairy and poultry environments [100]. These findings demonstrate the need for systematic cross-sectoral surveillance linking environmental signals with human and animal health outcomes.

While this narrative review provides an integrative overview of AMR across human, animal, and environmental sectors, it is limited by the inherent constraints of a narrative synthesis. The included studies were selected based on relevance rather than through a systematic, protocol-driven process, which may introduce selection bias and restrict reproducibility. In contrast, systematic reviews and meta-analyses offer quantitative assessments by following transparent inclusion criteria, critically appraising study quality, and statistically pooling data to derive more precise estimates of resistance prevalence or intervention efficacy. Such quantitative approaches allow for identification of heterogeneity among studies, trend estimation, and stronger evidence-based recommendations. The present review, therefore, should be viewed as a broad contextual framework that synthesizes multidisciplinary perspectives, while future systematic and meta-analytic studies are needed to validate the relationships and patterns highlighted here and to generate robust, comparable estimates of AMR burden across sectors and regions.

Finally, while global initiatives such as the WHO Global Action Plan, FAO/WOAH frameworks, and the O’Neill Report set a clear strategic agenda, practical implementation remains uneven. Barriers include insufficient enforcement of antimicrobial regulations, limited financial and human resources, entrenched practices such as over-the-counter antibiotic sales, and political fragmentation between ministries overseeing human health, agriculture, and the environment. Addressing these systemic challenges is critical to translating international frameworks into tangible impact at regional and national levels.

## 4. Conclusions

AMR is a complex, multifactorial challenge that demands urgent, coordinated action across human, animal, and environmental sectors. Moving forward, strengthening antimicrobial stewardship in both human and veterinary medicine is essential, with strict regulation of non-therapeutic use, wider adoption of rapid diagnostics, and consistent enforcement of prescription-only policies. At the same time, scaling up integrated One Health surveillance systems that capture resistance trends across hospitals, farms, wastewater, and wildlife especially in (LMICs) where data remain scarce will be critical. Equally important is investment in sustainable alternatives and technologies, including vaccines, probiotics, advanced wastewater treatment, and improved manure management, to reduce reliance on antibiotics and limit environmental contamination. Finally, empowering communities through farmer training, healthcare worker education, and public awareness campaigns, supported by international collaboration and robust policy frameworks, will provide the social foundation needed to sustain long-term AMR containment.

## Data Availability

No new data were created or analyzed in this study. Data sharing is not applicable to this article.

## Abbreviations

The following abbreviations are used in this manuscript:

AGPs	Antimicrobial Growth Promoters
AMR	Antimicrobial Resistance
APIS	Active Pharmaceutical Ingredients
ARB	Antimicrobial-Resistant Bacteria
ARGs	Antimicrobial Resistance Genes
AROs	Antibiotic-Resistant Organisms
CRE	*Carbapenem-resistant Enterobacteriaceae*
DOAJ	Directory of open access journals
ESBL	Extended-Spectrum Beta-Lactamase
FAO	Food and Agriculture Organization
HAIs	Healthcare-Associated Infections
HGT	Horizontal Gene Transfer
LD	Linear dichroism
MDPI	Multidisciplinary Digital Publishing Institute
MDR	Multidrug-Resistant
MRSA	Methicillin-Resistant Staphylococcus aureus
PMQR	Plasmid-Mediated Quinolone Resistance
TLA	Three-letter acronym
WHO	World Health Organization
WOAH	World Organisation for Animal Health
WWTP	Wastewater Treatment Plant
